# Rheumatoid arthritis patients initiating rituximab with low number of previous bDMARDs failures may effectively reduce rituximab dose and experience fewer serious adverse events than patients on full dose: a 5-year cohort study

**DOI:** 10.1186/s13075-022-02826-6

**Published:** 2022-06-02

**Authors:** Antonios Bertsias, Nestor Avgoustidis, Ioannis Papalopoulos, Argyro Repa, Nikolaos Kougkas, Eleni Kalogiannaki, Georgios Bertsias, Irini Flouri, Prodromos Sidiropoulos

**Affiliations:** 1 Rheumatology, Clinical Immunology and Allergy Department, Medical School University of Crete, 71110 Voutes, Heraklion, Greece; 2 Institute of Molecular Biology and Biotechnology, Foundation for Research and Technology Hellas (FORTH), Heraklion, Greece

**Keywords:** Rheumatoid arthritis, Rituximab, Observational study, Taper

## Abstract

**Background:**

Rituximab is used for the treatment of active rheumatoid arthritis. In the present study, we examined the long-term flare risk and safety of reduced doses of rituximab.

**Patients-methods:**

This was a prospective, observational, single-center study of patients starting rituximab on standard dose (SD). Patients were switched to low dose (LD) (1 g every 6 months), based on the treating rheumatologist’s decision after having achieved sustained clinical responses, while the rest of the patients continued on standard dose (SD). During a 60-month period, we assessed (Kaplan–Meier survival analysis) the relapse rate (increase ≥ 1.2 in DAS28-ESR for ≥ 6 months) and discontinuations due to treatment failure in the low dose group, and we compared the incidence of serious adverse events (SAEs) between LD and SD groups.

**Results:**

Out of 361 patients [females 83.4%, mean age 61.9 (10.6) years, seropositive 50.3%, median total comorbidities count 4], 81 patients (22.4%) entered LD in a median time of 24 months (95% CI 18–30 months). Seropositivity (OR 1.823), more than 2 previous bDMARDs failures (OR 0.428), and DAS28 < 4.88 at 6 months (OR 2.329) predicted the odds of entering LD (*p* < 0.05 for all). During 60 months of follow-up, only 7.5% of patients on LD relapsed. Patients on LD had significantly less SAEs and all-cause hospitalizations as compared to the SD group (*p* < 0.05 for all). Linear regression analysis showed that previous hospitalization while on bDMARDs (*p* < 0.0001), use of prednisolone > 5 mg/day while on rituximab (*p* < 0.0001), and a history of ≥ 2 previous csDMARDs (*p* = 0.041) predicted the risk of SAEs.

**Conclusion:**

In a cohort of patients with established RA and significant comorbidities who taper rituximab after substantial initial disease activity improvement, a low rate of relapses and lower risk of SAEs compared to SD were recorded. Seropositivity, a lower number of previous bDMARDs use, and lower DAS28 at 6 months predicted the probability of entering the LD regimen.

**Supplementary Information:**

The online version contains supplementary material available at 10.1186/s13075-022-02826-6.

## Background

Rituximab is among the biologic agents approved for the treatment of active rheumatoid arthritis (RA) not responding or intolerant to conventional synthetic disease-modifying drugs (csDMARDs) or biologic DMARDs (bDMARDs) [1]. The approved dosing regimen is 1000 mg for two consecutive infusions 2 weeks apart. The need for further courses is recommended to be evaluated 24 weeks following the initial course. Randomized controlled trials (RCTs) of rituximab in methotrexate (MTX)-inadequate responders and MTX-naive patients, showed comparable efficacy of reduced doses of RTX (two infusions of 500 mg) to that of standard doses in terms of reduction of disease activity [2–4]. Moreover, a meta-analysis of RCTs and cohort studies comparing low-dose (1000 mg) to “standard dose” of RTX (2000 mg) found similar effectiveness and advocated the use of the low-dose regimen, considering cost benefits [5]. The practice of tapering bDMARDs in RA patients on sustained remission or low disease activity, has been proved in several clinical trials mostly for tumor necrosis factor (TNF) inhibitors and it is proposed by current treatment guidelines [1]. Lowering the cost of RA treatment and reducing long-term cumulative bDMARDs doses is the main reasons supporting this practice.

In the present observational study, we evaluated the long-term effectiveness and safety of reduced doses of rituximab in a large cohort of RA patients of clinical practice, who achieved clinical responses while on initial standard doses of rituximab.

## Methods

We analyzed data from the University of Crete Rheumatology Clinic Registry (UCRCR), a single-center prospective cohort study initiated in 2004. According to the protocol—and after their informed consent—patients with inflammatory arthritis are included in the registry at the time they start their first bDMARD. Demographics, comorbidities assessed both as comorbidities count (CC) and based on the “Rheumatic Disease Comorbidities Index” (RCDI), disease characteristics, and extra-articular manifestations, as well as disease activity and function indices, are recorded at baseline. Patients are followed-up every 3–6 months with disease activity [disease activity score using 28 joints (DAS28), 28 swollen joint count (28SJC), 28 tender joint count (28TJC), 44SJC, 44TJC, visual analog score (VAS) global, VAS pain, VAS physician, erythrocyte sedimentation rate (ESR) and C-reactive protein (CRP)], function [modified Health Assessment Quaestionnaire (mHAQ)], and quality of life (EQ-5D) indices in every follow-up visit. Detailed data regarding rheumatologic drugs and their dosages, as well as treatment discontinuations and all adverse events (based on MedDRA coding) during follow-up, are also recorded.

Since 2015, patients who achieve remission or low disease activity for ≥ 2 consecutive 3-month visits or who have a sustained significant improvement (DAS28 decrease > 1.2) from baseline and, based on the clinical judgment of the treating rheumatologist would be benefited from rituximab tapering, enter the low dose (LD; 1 g/6 months) scheme. All other patients continue standard treatment and follow-up on standard dose (SD; 2 g). For the present study we included all patients in UCRCR who received rituximab and entered LD or continued in SD scheme. In patients who entered the LD regimen, flare was defined as the increase in DAS28 from LD start of ≥ 1.2 for 2 consecutive 3-month visits.

All demographic and baseline characteristics of patients were presented using descriptive statistics. Between-group comparisons were performed using Pearson’s chi-square tests or Fisher’s exact test in cases of categorical variables. Between-group comparisons were also performed using independent samples *T*-test or Mann–Whitney *U* test in cases of normally or non-normally distributed numeric variables respectively. A Kaplan–Meier survival analysis was performed using flare (yes/no) as the event variable and time of flare as the time-to-event variable. Non-parametric pairwise comparisons were performed using Wilcoxon signed-ranked test. A repeated-measures linear mixed model was performed with the dependent variable as the DAS28-ESR score and the dosage group (low dose vs high dose) as the independent variable. A multi-variable linear regression model was performed using the number of serious adverse events as an independent variable and gender (male/female), age, RCDI, the number of concomitant non-biologic medications, the number of previous biologics (≥ 2 biologics versus < 2 biologics), the concomitant prednisolone > 5 mg/day (yes/no), the past hospitalization for serious infection (yes/no) and the study group (low dose vs high dose) was performed. A multi-variable logistic regression model was performed using as dependent variable the dose scheme (low dose vs high dose) and independent variables gender (male vs female), age, disease duration (in years), the RCDI, patient being seropositive (yes/no), the number of previous biologics (> 2 biologics vs ≤ 2 biologics), the DAS28-ESR scores at baseline, and the DAS28-ESR category levels at 6 months which resulted upon median split (≥ 4.88 versus < 4.88). An intention-to-treat analysis was carried throughout. The level of statistical significance was set to *α* = 0.05 and the statistical software that was used was IBM SPSS version 25 and STATA SE 11.

## Results

### Patients’ characteristics

We studied 361 patients who started rituximab according to the treating rheumatologist’s decision following the national and EULAR guidelines for the treatment of RA. Patients have mostly established RA [median disease duration 5 years (min 0.5, max 51; IQR 9 years)], half of them were seropositive for rheumatoid factor (RF) or anti-CCP antibodies (ACPA) and received rituximab as the third (median) bDMARD treatment (min = 1, max = 7) (Table 1). After a median time of 24 months (95% CI for the median 18–30 months) therapy with the standard dose of rituximab, 81 patients (22.4%) entered the LD scheme. Forty-five out of the 81 (55.5%) patients satisfied the “criterion” of sustained remission or low DAS28 score (DAS28 < 3.2). An additional 28.4% (*n* = 23) of the patients had tender or swollen joint count $\le$ 3 while having high patient’s VAS global (> 60), and were considered as having “well-controlled disease” according to their rheumatologist’s opinion. The remaining 14% switched to the LD scheme due to older age/frailty and patient’s choice. Patients on LD were more often seropositive for RF or ACPAs (*p* = 0.023 and *p* = 0.016, respectively) and received rituximab earlier as a bDMARD compared to patients remaining on SD (RTX as ≤ 2nd treatment bDMARD line vs ≥ 3rd line, *p* = 0.023). All patients have had a prior exposure to a high number of csDMARDs, (methotrexate: 90.9%, leflunomide: 64.8%), while csDMARDs history as well as prior steroids’ exposure (63.4% on prednisolone ≤ 10 mg/day) were comparable among SD and LD groups (Supplementary Table 1). Furthermore, 77.0% were exposed to anti-TNFα agents and 33.8% to non-TNFα inhibitors prior to starting rituximab.

**Table 1:** Baseline characteristics of the cohort. Univariate comparisons between patients under SD and LD rituximab

	**All patients (*n* = 361)**	**Standard dose (*n* = 280)**	**Low dose (*n* = 81)**	***p* value**
**Gender** (female)	301 (83.4%)	234 (83.6%)	67 (82.7%)	0.855
**Age (years),** mean (SD)	61.9 (10.6)	62.3 (10.4)	60.3 (11.5)	0.130
**Seropositive**	171 (50.3%)	97 (44.7%)	49 (67.1%)	0.001
Rheumatoid factor positive	134 (40.2%)	94 (36.9%)	40 (51.3%)	0.023
ACPA positive	124 (42.0%)	84 (38.0%)	34 (45.9%)	0.016
**Disease duration, **median (min, max, IQR)	5 (1, 51; 9)	5 (1, 51; 9)	6 (1, 37; 8)	0.031
**RTX biologic treatment number,** median (min, max; IQR)	3 (1, 7; 2)	3 (1,7; 2)	2 (1,6; 2)	0.023
**Ever smoker**	101 (41.7%)	70 (39.1%)	31 (49.2%)	0.162
**Co-morbidities**				
**RCDI, median (min, max; IQR)**	2 (0, 7; 3)	2 (0,7;3)	2 (0,7;4)	0.369
**Total number of comorbidities, median (min, max; IQR)**	4 (0, 15; 7)	4 (0, 15; 7)	4 (0; 14; 6)	0.613
**Circulatory-metabolic**				
Hypertension	166 (46.0%)	135 (48.2%)	31 (38.3%)	0.114
Dyslipidemia	159 (44.0%)	124 (44.3%)	35 (43.2%)	0.864
Obesity	143 (39.6%)	110 (39.3%)	33 (40.7%)	0.814
Type-II diabetes	66 (18.3%)	51 (18.2%)	15 (18.5%)	0.950
Hypothyroidism	84 (23.3%)	60 (21.4%)	24 (29.6%)	0.124
Ischemic stroke TIA	12 (3.3%)	11 (3.9%)	1 (1.2%)	0.313
**Cardiac**				
Valvular heart disease	30 (8.3%)	18 (6.4%)	12 (14.8%)	0.016
Coronary heart disease	28 (7.8%)	19 (6.8%)	8 (9.9%)	0.352
Cardiac arrythmia	18 (5.0%)	14 (5.0%)	4 (4.9%)	0.981
Congestive heart failure	17 (4.7%)	11 (3.9%)	6 (7.4%)	0.193
**Pulmonary**				
COPD	27 (7.5%)	18 (6.4%)	9 (11.1%)	0.158
Asthma	20 (5.5%)	17 (6.1%)	3 (3.7%)	0.583
**Psychiatric**				
Depression	71 (19.7%)	63 (22.5%)	17 (20.1%)	0.352
Anxiety disorder	15 (4.2%)	10 (3.6%)	5 (6.2%)	0.301
**Infectious history**				
Hospitalization for serious infection	84 (23.3%)	65 (23.2%)	19 (23.5%)	0.964
Recurrent hospitalizations for serious infections	29 (8.0%)	24 (8.6%)	5 (6.2%)	0.484
Latent TB	46 (12.7%)	40 (14.3%)	6 (7.4%)	0.102
Viral hepatitis (HCV/past HBV)	13 (3.6%)	8 (2.9%)	5 (6.2%)	0.158
Past herpes zoster	8 (2.2%)	4 (1.4%)	4 (4.9%)	0.079
**Cancers**				
Hodgkin lymphoma	6 (1.7%)	5 (1.8%)	1 (1.2%)	0.733
Non-Hodgkin lymphoma	3 (0.8%)	1 (0.4%)	2 (2.5%)	0.128
Leukemia	4 (1.1%)	3 (1.1%)	1 (1.2%)	0.902
Solid tumor	23 (6.4%)	13 (4.6%)	5 (6.2%)	0.567

A significant burden of comorbidities was recorded at the treatment baseline [median total comorbidities count (CC): 4; median RDCI: 2], comparable between the 2 groups (Table 1). As expected, hypertension and dyslipidemia were the most commonly reported comorbidities (46% and 44% of patients respectively). Interestingly, 23.3% and 8% of the patients had a history of hospitalization or recurrent hospitalizations for serious infection respectively while on previous bDMARDs, comparable between the two groups. Ten patients had “past HBV”, 2 HCV infections, and one patient concomitant HCV and past HBV [anti-HBc ( +), anti-HBs ( −)], while 8 had a history of past reactivation of varicella-zoster virus during treatment with a previous biologic agent. During follow-up, we observed no new cases of hepatitis or HBV reactivation in patients with past infection in either group. Finally, 13 (3.6%) and 23 (6.4%) patients had a history of hematologic or solid tumor malignancy respectively.

### Disease characteristics at rituximab initiation and predictors of entering the LD group

At rituximab initiation, patients had highly active RA [median DAS28 5.73 (1.74, 8.69; 1.43)] and compromised functionality [median mHAQ 1.0 (0, 2.8; 0.8)], comparable between LD and SD groups (Table 2). Patients eventually entering the LD regimen had higher baseline inflammatory markers, both ESR and CRP (*p* = 0.003 and *p* = 0.006, respectively), while patients on SD had more swollen joints (*p* = 0.015). Rituximab was given as monotherapy in 15.2% of the patients, while 61.5% were on combination with MTX and 45.1% on prednisolone (Table 2). Concomitant RA-related medications were comparable between the two groups. Logistic regression analysis revealed that seropositivity (for RF or ACPAs) [OR 1.823 (1.009–3.292), *p* = 0.046], past use of > 2 bDMARDs [OR 0.428 (0.204–0.898, *p* = 0.025] and a DAS28 lower than 4.88 at 6 months [OR 2.329 (1.254–4.325), *p* = 0.007] predicted entering the LD group (Table 3).

**Table 2:** Disease characteristics and co-administered medications at rituximab initiation (univariate comparisons between patients under SD and LD RTX)

	**All patients**	**Standard dose**	**Low dose**	***p* value**
**RA-related characteristics**				
DAS28-ESR	5.73 (1.74, 8.69; 1.43)	5.77 (1.93, 8.69; 1.42)	5.66 (1.74, 8.66; 1.590	0.800
DAS28-CRP	4.70 (1.06, 7.32; 1.31)	4.70 (1.07, 7.02; 1.19)	4.66 (1.06, 7.32; 1.49)	0.279
ESR	29 (0, 135; 28)	27 (0, 135; 29)	34 (3, 92; 28)	0.003
CRP (mg/dL)	0.34 (0, 14; 0.64)	0.33 (0.00, 14.00, 0.50)	0.46 (0.00, 13.40; 1.64)	0.006
Swollen 28	8 (0, 27; 7)	9 (0, 27; 7)	7 (0, 24; 6)	0.015
Tender 28	9.5 (0, 28; 10)	10 (0, 28; 11)	8 (0, 26; 11)	0.098
VAS Global	70 (0,100; 30)	70 (0, 100; 30)	70 (0,100;30)	0.949
VAS Phys	75 (5,100; 12)	75 (5, 100; 10)	75 (25, 100; 25)	0.058
VAS pain	70 (0,100; 30)	70 (0, 100; 30)	70 (20, 100; 25)	0.995
mHAQ	1.0 (0, 2.8; 0.8)	1.0 (0, 2.8; 0.7)	1.0 (0, 2.1; 1.0)	0.360
EQ5D	0.175 (-0.43, 0.80; 0.66)	0.2 (-0.43, 0.80; 0.61)	0.0 (-0.24, 0.73; 0.67)	0.217
**Co-medications**				
MTX	222 (61.5%)	164 (58.6%)	58 (71.6%)	0.120
Leflunomide	106 (29.4%)	82 (29.3%)	24 (29.6%)	0.952
Hydroxychloroquine	71 (19.7%)	53 (18.9%)	18 (22.2%)	0.511
Sulfasalazine/cyclosporine	7 (2%)	6 (2.1%)	1 (1.2%)	0.895^a^
Prednisolone	163 (45.1%)	125 (44.6%)	38 (46.9%)	0.756
Prednisolone daily dose, median (min, max, IQR)	5 (2.5, 5, 5)	6 (2.5, 5, 5)	5 (2.5, 20, 5)	0.638
RTX monotherapy	55 (15.2%)	46 (16.4%)	9 (11.1%)	0.241

^a^Fisher’s exact test

**Table 3:** Logistic regression analysis predicting the odds of entering the low-dose regimen

Predictor	Odds ratio	95% confidence interval	*p* value
Gender	0.809	0.504 to 2.405	0.809
Age	0.974	0.945 to 1.004	0.084
Disease duration (years)	1.011	0.978 to 1.046	0.511
RCDI	1.112	0.948 to 1.305	0.193
Seropositive (yes)	1.823	1.009 to 3.292	0.046
> 2 previous non-biologics	1.259	0.688 to 2.306	0.455
> 2 previous biologics (yes)	0.428	0.204 to 0.898	0.025
DAS28-ESR start	0.969	0.750 to 1.252	0.810
DAS28-ESR 6 months < 4.88 (yes)	2.329	1.254 to 4.325	0.007

Chi-square 25.399; *p* = 0.003 on 9 degrees of freedom; adjusted *R*^2^ = 0.131

### Flare risk during follow-up in the low dose group

After a median time of treatment with SD of 24 months (95% CI 18–30 months), 81 (22.4%) patients tapered rituximab to 1 g/6 months. Disease activity in both SD and LD groups improved significantly during the first 24 months of treatment from 5.77 (1.93, 8.69; 1.42) and 5.66 (1.74, 8.66; 1.59) to 4.56 (1.81,7.38;1.96), and 3.76 (1.54, 7.80;1.94), respectively, (*p* < 0.0001 for both). As expected, those who tapered had a significantly higher improvement at 6 months compared to baseline [median DAS28 difference 0.65 (− 3.00, 4.20; 1.64) for the SD and 1.11 (− 2.20, 4.54; 1.78) for the LD (*p* = 0.017)].

**Fig. 1:**
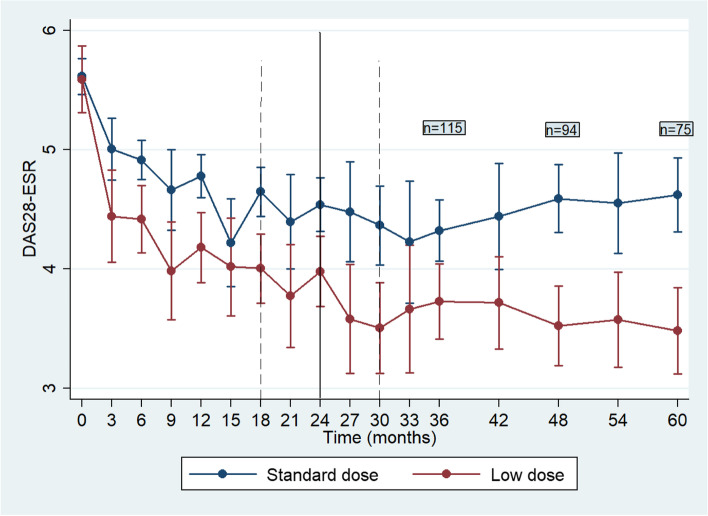
Linear mixed model predictions of DAS28-ESR score according to rituximab dosing group

**Fig. 2:**
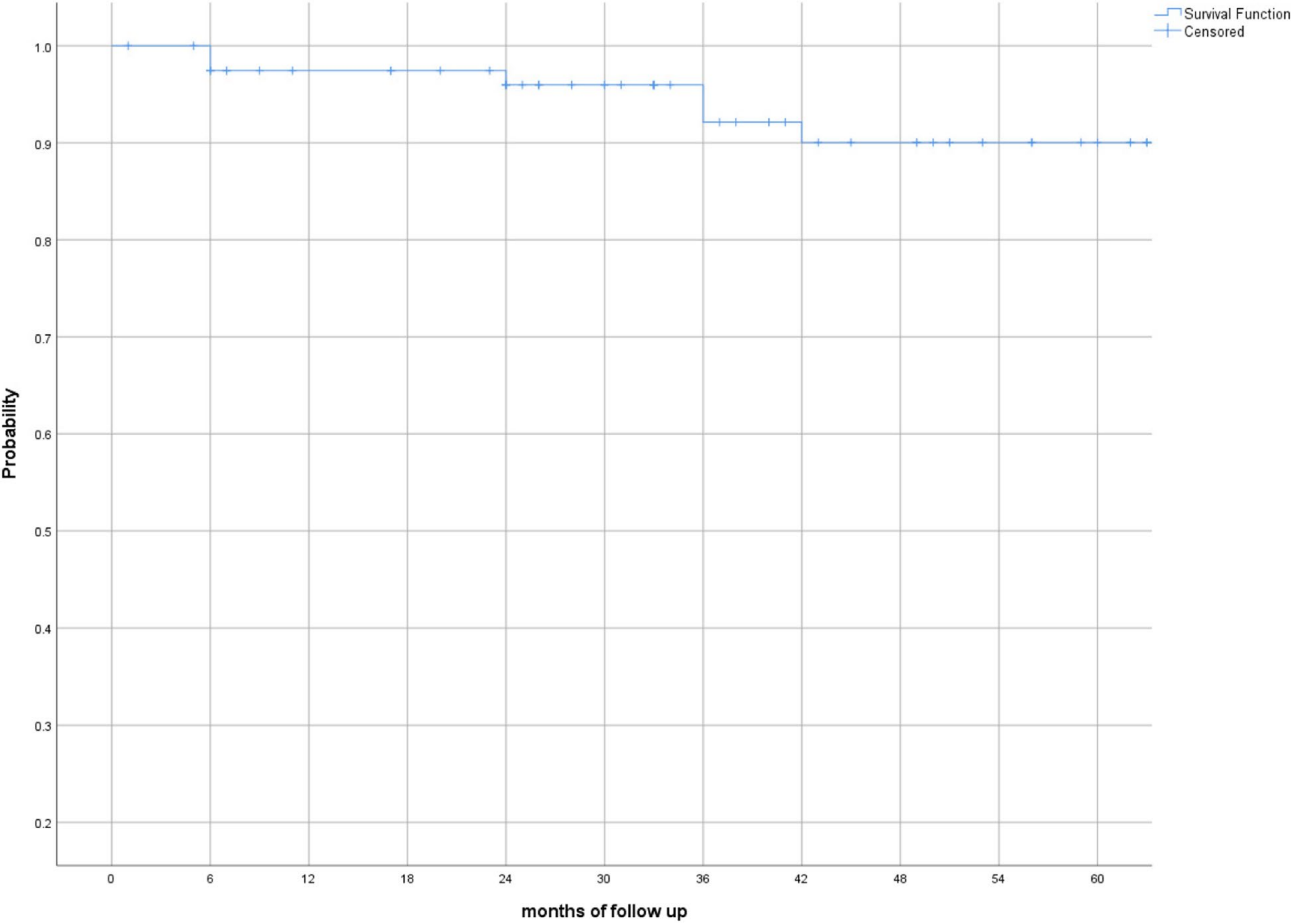
Survival plot of flare probability within the low dose group

After rituximab tapering, patients were prospectively followed for a median of 56 (1, 177; 59) months. For the LD group, DAS28 ESR at last visit [median 3.33 (1.05, 7.82; 2.12)] was comparable to DAS28 at rituximab taper baseline visit [median 3.28 (1.05, 6.58;1.88)] (*p* = 0.639) (Fig. 1). We further assessed for disease flares in both groups. During 60 months of follow-up, only 5.9% and 7.5% of the patients in SD and LD, respectively, experienced a flare of RA (*p* = 0.6) (Table 4). The K-M survival analysis (having as event a flare of the disease), is given in Fig. 2. Moreover, and in order to assess the clinical utility of the tapering strategy, we assessed for discontinuations due to treatment failure. We found that patients on LD had significantly less discontinuations due to failure as compared to those on SD (37.9% vs 63.6%, respectively, *p* < 0.0001) (Table 4). A total of 18 of 177 (10.2%) discontinuations were due to infections. Interestingly, 3 out of 18 (16.7%) patients who discontinued due to infections had low IgG levels (two in the standard dose group, one in the low dose group).

**Table 4:** Drug exposure and rituximab discontinuation reasons

	**All patients (*n* = 361)**	**Standard dose (*n* = 280)**	**Low dose (*n* = 81)**	***p* value**
Total follow-up time (months), median (min, max, IQR)	19 (1, 177; 46)	14.5 (1, 127; 27)	56 (1, 177; 59)	< 0.0001
Total cumulative RTX exposure (g), median (min, max, IQR)	38 (2, 254; 72)	28 (2, 254; 52)	90 (2, 246; 78)	< 0.0001
Disease flares	22 (6.3%)	16 (5.9%)	6 (7.5%)	0.599
**Reasons for RTX discontinuation**				
**Failures**^a^	105 (59.3%)	94 (63.6%)	11 (37.9%)	< 0.0001
**Adverse events as stop reason**^b^ **(all)**	34 (19.2%)	25 (16.9%)	9 (31.0%)	0.131
*Infections*	18 (10.2%)	14 (9.5%)	4 (13.8%)	0.711
*Other adverse events*	16 (9.0%)	11 (7.4%)	5 (17.2%)	0.183
**Other reasons**^c^	38 (21.5%)	29 (19.5%)	9 (31.1%)	0.002

^a^Includes primary and secondary failures

^b^Includes all adverse events which led to rituximab discontinuation

^c^Includes financial, practical, patient decision, pregnancy, frailty, remission

### Safety and events

We followed LD patients for a median of 56 (1, 177; 59) months, total period of 7287 person-months, while patients on SD were followed up for 14.5 (1, 127; 27) months or 4824 person-months (Table 4). Lower follow-up period was due to a higher discontinuation rate of patients on SD (52.9%) as compared to that of patients on LD (35.8%). Patients on LD were cumulatively exposed to 90 g (2, 246; 78) of rituximab as compared to 28 g (2, 254; 52) in SD groups. During this period, a total of 45 and 137 serious adverse events (SAEs) in LD and SD respectively accounting for an incidence rate of 0.77 and 1.57 events per 1000 person-years, were documented (*p* < 0.0001) (Table 5). Incidence rates for serious infections (0.49 vs 0.88, *p* = 0.0026) and all-cause hospitalizations (0.53 vs 1.08, *p* = 0.0002), were lower in LD than in SD group. As a sensitivity analysis, given the heterogeneity and the differences of rituximab exposure between SD and LD groups, we also compared adverse events between more “homogenous” subgroups of SD and LD. Thus, we compared the incidence rates of adverse events (events per 1000 person-years) for those patients with a total follow-up time of ≥ 24 months in both groups. In accordance to the findings of the main analysis, a more favorable safety profile of LD as compared to the SD group was found for moderate/serious AE, serious infections, and hospitalizations (*p* < 0.001 for all, Supplementary Table 2).

**Table 5:** Comparison of incidence rates of adverse events (events per 1000 person-years) for all patients and by dose group

	**All patients**	**Standard dose**	**Low dose**	***p* value**
**Total person-months of follow-up**	12,111	4824	7287	
**Number of adverse events (moderate and serious)**	735	509	226	
Incidence rate for adverse events	5.07	5.82	3.90	< 0.0001
**Number of serious adverse events**	182	137	45	
Incidence rate of serious adverse events (grade IV–VI)	1.25	1.57	0.77	< 0.0001
**Number of serious infections**	103	75	28	
Incidence rate of serious infections	0.72	0.88	0.49	0.0026
**Number of all hospitalizations**	125	94	31	
Incidence rate for hospitalizations	0.86	1.08	0.53	0.0002
**Number of incident cancer cases**	12	8	4	
Incidence rate for cancer diagnosis	0.08	0.09	0.07	0.6719
**Number of incident deaths**	14	11	3	
Incidence rate for death	0.14	0.18	0.08	0.0994

We finally assessed for predictors of serious adverse events. Linear regression analysis showed that past hospitalization for serious infection while one a previous bDMARD (beta 0.974, *p* < 0.0001), sustained use for at least 6 months of prednisolone > 5 mg/day while on rituximab (beta 0.689, *p* < 0.0001) and > 2 previous csDMARDs use (beta 0.320, *p* = 0.041) were all independent predictors of a higher number of serious adverse events in the cohort (Table 6).

**Table 6:** Linear regression using as dependent variable the number of serious adverse events in the cohort

Predictor	Beta	95% confidence interval	*p* value
Gender	0.163	− 0.151 to 0.477	0.308
Age	− 0.003	− 0.014 to 0.009	0.651
RCDI	0.033	− 0.029 to 0.095	0.294
Number of concomitant non-biologics	− 0.108	− 0.291 to − 0.075	0.246
≥ 2 previous non-biologics (yes)	0.320	0.013 to 0.627	0.041
≥ 2 of previous biologics (yes)	− 0.096	− 0.331 to 0.140	0.425
Low dose (yes)	0.003	− 0.276 to 0.274	0.995
Prednisolone > 5 mg/day for at least 2 FUs in cohort	0.689	0.373 to 1.006	< 0.0001
Past hospitalization for serious infection (yes)	0.974	0.690 to 1.259	< 0.0001

F-statistic 9.823; *p* < 0.0001 on 9 degrees of freedom, *n* = 350 included in the analysis

## Discussion

In this observational study we found that in RA patients with initial sustained clinical response to standard doses of rituximab, reduced doses during a 5-year period have a low risk of disease’s flare and discontinuations due to treatment failure, while patients experienced significantly less serious adverse events as compared to patients who continue with standard doses. In our group of patients with established disease and a high comorbidities burden, predictors of entering the LD scheme as well as factors predicting serious events while on LD were identified.

Tapering bDMARDs has been a strategy suggested for RA patients with persistent clinical responses (mostly in remission) upon initially standard doses of bDMARDs [1]. Most of the data supporting this approach are based on trials of TNF inhibitors [6]. Controlled clinical trials of rituximab in standard and reduced doses have been shown to have comparable both biological effects (CD19 + ve B cells depletion/repopulation) and clinical responses [7]. Reduced doses of rituximab have been assessed either from the first dose or after initial standard doses [2–4, 7]. The above mentioned are randomized studies, with mostly a short follow-up period, except the study of Mariette et al. which has a follow-up of 104 weeks. Concerning the effect of reduced rituximab doses on joint damage protection, data from RCTs are not consistent, and one could argue that the protective effect on joint damage of standard compared to lower doses could be different. In MTX-naïve patients, a differential protective effect between standard and a low dose of rituximab after 1 year has been found [4]. Interestingly enough, the 2 years analysis of the IMAGE study showed a favorable protective effect of rituximab low dose [8]. Nevertheless, this study was not powered to compare rituximab doses and patients were MTX-naïve, a population for whom rituximab is not yet approved.

Of note, in clinical practice, the common scheme is that of the “standard dose” (1000 mg 2 times 15 days apart). Interestingly, a more recent observational, prospective study [Autoimmunity and Rituximab (AIR)] showed that after a first course of rituximab at standard dose patients that have been re-treated with low dose of RTX remained on therapy at 5 years and received reduced cumulatively doses of the monoclonal [9]. Our study and that of Henry et al., have the longest follow-up and comparable results. Both these observational studies showed that in the long term, low doses of rituximab have a high drug retention rate (53.8% in the French study), while in our study we documented only 7.5% relapse rate. In both studies, drug retention or frequency of relapses was comparable between low and standard doses of rituximab. Further supporting the value of the concept to taper rituximab in patients with the initial response, we found that only 37.9% vs 63.6% of patients discontinued therapy due to treatment failure on LD and SD respectively (*p* < 0.0001).

In our cohort of 361 RA patients, we aimed to identify factors at rituximab initiation which predict who will enter the LD group. Logistic regression analysis revealed that seropositivity (OR 1.823), past use of > 2 bDMARDs (OR 0.428), and a DAS28 lower than 4.88 at 6 months (OR 2.329) predicted entering the low-dose group (Table 3). In our study, patients who entered the low dose were responders to initial standard doses of rituximab, and thus the above predictors can be considered as factors predicting long-term clinical responses to rituximab. Seropositivity and a lower number of previous bDMARDs failures have been shown as predictors for response to rituximab in several studies [10–13]. We and others have shown that an early (during the first year of treatment) clinical response to TNFα inhibitors may predict anti-TNFα survival [14, 15]. Herein and comparable to the above anti-TNFα related data, we showed that an improvement in DAS28 during the first 6 months of treatment may predict long-term rituximab drug survival. All the above factors were predictors of long-term rituximab efficacy and drug survival, and we consider this as a clinically important finding of our analysis.

The second main finding of our study was that the LD group experienced a significantly lower number of all adverse events, SAE, and hospitalizations as compared to SD group (Table 5). Our findings are in line with the results reported by Henry et al., showing a lower rate of serious infections in the reduced dose group [9]. Both studies, as mentioned above, are the observational studies with the longest follow-up of patients in everyday clinical practice. Interestingly, a numerically lower risk of serious infections in patients on reduced doses of RTX has been reported in a meta-analysis of randomized controlled trials (RCTs) comparing standard and reduced doses (0.73 (0.37–1.47), *p* = 0.38) [5]. Patient selection and the short-term follow-up of the patients in RCTs may explain differences between observational studies and the aforementioned meta-analysis. We further explored for predictors of serious adverse events. Interestingly, we found that past hospitalization for serious infection while one a previous bDMARD as the most important risk factor for SAE. This is a novel finding not reported in the literature before. Since our registry is focused in all patients starting the first bDMARD, we were able to analyze prior history (while on bDMARDs) of AE and medications as predisposing factors for SAE while on rituximab. As expected, sustained use for at least 6 months of prednisolone > 5 mg/day while on rituximab was also an independent risk factor for SAE, underling the importance of tapering/stopping steroids in order to minimize the risk for SAE in our patients. The association of low doses of steroids with adverse events (mostly infections) has been reported multiple times in different RA cohorts under multiple therapies [14, 16, 17].

This was an observational, single-center study, of patients followed by a standard protocol. Patients’ selection for tapering rituximab was allowed to be the treating physician’s decision, based on clinical responses and patient profile and not based on a standard protocol. Thus, 44 out of the 81 (55.5%) patients satisfied the “criterion” of sustained remission or low DAS28 score (DAS28 < 3.2), while an additional 28.4% (*n* = 23) of the patients had tender or swollen joint count $\le$ 3 while having high patient’s VAS global (> 60), and were considered as having “well-controlled disease” according to their rheumatologist’s opinion. Moreover, although disease activity in both SD and LD groups improved significantly during the first 24 months of treatment, those who tapered had a significantly higher improvement at as early as 6 months [median DAS28 difference 0.65 (− 3.00, 4.20; 1.64) for the SD and 1.11 (− 2.20, 4.54; 1.78) for the LD (*p* = 0.017)]. Thus, we consider that although we do not follow a strict protocol for tapering rituximab, patients who entered the LD scheme were those with significant improvement in disease activity after initial standard doses of rituximab.

One could argue that the practice of continuing rituximab for those patients not achieving “treat-to-target” goals is not acceptable in clinical practice. Nevertheless, SD patients had clinically and statistically important improvement in disease activity during the first 24 months of treatment from 5.77 (1.93, 8.69; 1.42) to 4.56 (1.81,7.38;1.96) (*p* < 0.0001). Moreover, as pointed out in cohort characteristics, patients on SD had established, active RA after a failure of a median of 2 (up to 6) bDMARDs and 2 (up to 7) of csDMARDs with a high burden of comorbidities 4 (up to 15). We consider that treatment switches in a cohort like this, are not strictly based on the “treat-to-target” approach; both patients and rheumatologists are considering several other factors for treatment decisions.

The percentage of rheumatoid factor or anti-CCP antibodies positivity was relatively low in our cohort as compared to other observational studies [9]. Given the data from RCTs and observational studies supporting a higher response rate of rituximab in seropositive patients, many clinicians apply seropositivity as a “biomarker” favoring rituximab among other bDMARDs. Nevertheless, a metanalysis of RCTs showed a modest differential effect in seropositive patients [18] and in observational studies, despite the higher responses in seropositive patients, clinically and statistically important improvements were found also in seronegative patients [10]. Moreover, the seronegative RA population is increased during the last decade compared to earlier studies [19]. Finally, data supporting that seropositive respond better than seronegative patients have been also published for TNFα inhibitors, abatacept, and tocilizumab [11, 20]. The above could explain the lower percentage of seropositive patients in our group as compared to other cohorts.

The two groups were heterogenous regarding their baseline characteristics, time of follow-up, and exposure to rituximab. Even though this is common in observational studies, it raises concerns for the interpretation of the findings. Nevertheless, we have to emphasize that SD group was a “control” group to LD only in terms of comparing the safety of the two groups and not for long-term clinical efficacy. Moreover, the sensitivity analysis of the rate of adverse events restricted to patients with a total follow-up time of ≥ 24 months, revealed a better safety profile of LD, similarly to the whole group analysis (Supplementary Table 2). Finally, although patients on LD had a higher exposure to rituximab compared to SD (90 vs 28 g) and longer time on rituximab (56 vs 14.5 months), yet they had a lower incidence of serious/moderate AE, lower incidence of serious infections and hospitalizations (Table 5). Thus, although and as expected due to the observational nature of the study, there were differences between the two groups, we consider the results of the comparisons clinically important, with a special focus on the safety of LD.

## Conclusion

Our prospective 5-year analysis of established RA patients with multiple bDMARDs failures and high comorbidities burden confirms that reduced doses of rituximab is a valid approach for patients with initial sustained clinical response. Patients starting rituximab after a failure to less than 2 bDMARDs, who are seropositive and improve disease activity at 6 months while on rituximab are those who have a higher probability to enter the LD. Rheumatologists should be aware of the increased risk of SAE in patients with a prior history of hospitalization and sustained use of steroids.

## Supplementary Information

**Additional file 1: Table S1.** Previous anti-rheumatic medications, and uni-variate comparisons between previous anti-rheumatic medication of patients under SD or LD RTX. **Table S2.** Comparison of incidence rates of adverse events (events per 1000 person-years) for all patients and by dose group for patients with a total follow-up time ≥24 months.

## Data Availability

The data and analytic methods that support the findings of this study are available to qualified investigators by request to the corresponding author.

## Abbreviations

SD: Standard dose
LD: Low dose
ESR: Erythrocyte sedimentation rate
SAEs: Serious adverse events
bDMARDs: Biologic disease-modifying anti-rheumatic drugs
csDMARDs: Conventional synthetic disease-modifying anti-rheumatic drugs
RCTs: Randomized controlled trials
MTX: Methotrexate
RTX: Rituximab
TNF: Tumor necrosis factor
RA: Rheumatoid arthritis
UCRCR: University of Crete Rheumatology Clinic Registry
RCDI: Rheumatic Disease Comorbidities Index
DAS28: Disease activity score using 28 joints
28SJC: 28 Swollen joint count
28TJC: 28 Tender joint count
44SJC: 44 Swollen joint count
44TJC: 44 Tender joint count
VAS: Visual analog score
CRP: C-reactive protein
mHAQ: Modified Health Assessment Questionnaire
EQ-5D: Quality of life
MedDRA: Medical Dictionary for Regulatory Activities
EULAR: European League Against Rheumatism
RF: Rheumatoid factor
ACPA: Anti-CCP antibodies
TNF: Tumor necrosis factor
CC: Comorbidities count
AIR: Autoimmunity and rituximab
TIA: Transient ischemic attack
COPD: Chronic obstructive pulmonary disease
TB: Tuberculosis
IQR: Inter quartile range
