# Supplementary material for: Rheumatoid arthritis patients initiating rituximab with low number of previous bDMARDs failures may effectively reduce rituximab dose and experience fewer serious adverse events than patients on full dose: a 5-year cohort study
Source: Arthritis Res Ther. 2022 Jun 2;24:132. doi: 10.1186/s13075-022-02826-6 (PMC9161491; doi:10.1186/s13075-022-02826-6)
Supplement: Supplementary file 1 — Additional file 1: Table S1. Previous anti-rheumatic medications, and uni-variate comparisons between previous anti-rheumatic medication of patients under SD or LD RTX. Table S2. Comparison of incidence rates of adverse events (events per 1000 person-years) for all patients and by dose group for patients with a total follow-up time ≥24 months. [file 13075_2022_2826_MOESM1_ESM.docx]

**Supplementary Table 1**. Previous anti-rheumatic medications, and uni-variate comparisons between previous anti-rheumatic medication of patients under SD or LD RTX.

| **Medications** | **All patients** | **Full-dose** | **Low dose** | **p-value** |
| --- | --- | --- | --- | --- |
| **csDMARDs** |  |  |  |  |
| MTX (any) | 338 (93.6%) | 260 (92.9%) | 78 (96.3%) | 0.264 |
| Leflunomide | 234 (64.8%) | 182 (65.0%) | 52 (64.2%) | 0.894 |
| Hydroxychloroquine | 190 (52.6%) | 150 (53.6%) | 40 (49.4%) | 0.506 |
| Azathioprine | 14 (3.9%) | 13 (4.6%) | 1 (1.2%) | 0.207* |
| Sulfasalazine | 55 (15.2%) | 31 (11.1%) | 24 (29.6%) | <0.0001 |
| Cyclosporine | 44 (12.2%) | 27 (9.6%) | 17 (21.0%) | 0.006 |
| **All TNFi** | 278 (77.0%) | 217 (77.5%) | 61 (75.3%) | 0.680 |
| Infliximab | 109 (30.2%) | 80 (28.6%) | 29 (35.8%) | 0.212 |
| Adalimumab (Sc-inj) | 127 (35.2%) | 95 (33.9%) | 32 (39.5%) | 0.355 |
| Etanercept (Sc-inj) | 124 (34.3%) | 105 (37.5%) | 19 (23.5%) | 0.019 |
| Golimumab (Sc-inj) | 42 (11.6%) | 38 (13.6%) | 4 (4.9%) | 0.033 |
| Certolizumab (Sc-inj) | 22 (6.1%) | 16 (5.7%) | 6 (7.4%) | 0.575 |
| **Non-TNFi** | 122 (33.8%) | 107 (38.2%) | 15 (18.5%) | 0.001 |
| Abatacept (IV + inj) | 86 (23.8%) | 74 (26.4%) | 12 (14.8%) | 0.031 |
| Tocilizumab (IV + inj) | 75 (20.8%) | 66 (23.6%) | 9 (11.1%) | 0.015 |
| Anakinra (Sc-inj) | 16 (4.4%) | 15 (5.4%) | 1 (1.2%) | 0.112 |
| Tofacitinib | 2 (0.6%) | 2 (0.7%) | 0 (0.0%) | 0.446* |
| Prednizolone ≤10 mg/day | 229 (63.4%) | 175 (62.5%) | 54 (66.7%) | 0.493 |
| Number of previous non-biologic medications Median (min, max; IQR) | 2 (0,7;1) | 2 (0,7;1) | 3 (1,6;2) | 0.099 |
| Number of previous biologic medications Median (min, max; IQR) | 2 (0,6;2) | 2 (0,6;2) | 1 (0,5;2) | 0.025 |
| *Fisher’s exact test | | | | |

**Supplementary Table 2**. Comparison of incidence rates of adverse events (events per 1000 person-years) for all patients and by dose group for patients with a total follow-up time ≥24 months

|  | **All patients**  **N=162** | **Standard dose**  **N=93** | **Low dose**  **N=69** | **p-value** |
| --- | --- | --- | --- | --- |
| **Total person-months of follow-up** | 10104 | 5404 | 4700 |  |
| **Number of adverse events (moderate & serious)** | 582 | 368 | 214 |  |
| Incidence rate for adverse events | 5.76 | 6.81 | 4.55 | <0.0001 |
| **Number of serious adverse events** | 135 | 93 | 42 |  |
| Incidence rate of serious adverse events (grade IV-VI) | 1.33 | 1.72 | 0.89 | 0.0001 |
| **Number of serious infections** | 80 | 55 | 25 |  |
| Incidence rate of serious infections | 0.79 | 1.03 | 0.53 | 0.0025 |
| **Number of all hospitalizations** | 88 | 62 | 26 |  |
| Incidence rate for hospitalizations | 0.87 | 1.14 | 0.55 | 0.0006 |
| **Number of incident cancer cases** | 9 | 5 | 4 |  |
| Incidence rate for cancer diagnosis | 0.89 | 0.85 | 0.92 | 0.456 |
| **Number of incident deaths** | 6 | 4 | 2 |  |
| Incidence rate for death | 0.08 | 0.06 | 0.01 | 0.302 |
